# Care Outcomes Among Black or African American Persons with Diagnosed HIV in Rural, Urban, and Metropolitan Statistical Areas — 42 U.S. Jurisdictions, 2018

**DOI:** 10.15585/mmwr.mm7007a1

**Published:** 2021-02-19

**Authors:** Shacara Johnson Lyons, André F. Dailey, Chenchen Yu, Anna Satcher Johnson

**Affiliations:** ^1^Division of HIV/AIDS Prevention, National Center for HIV/AIDS, Viral Hepatitis, STD, and TB Prevention, CDC; ^2^ICF, Atlanta, Georgia.

During 2018, Black or African American (Black) persons accounted for 43% of all diagnoses of human immunodeficiency virus (HIV) infection in the United States (*1*). Among Black persons with diagnosed HIV infection in 41 states and the District of Columbia for whom complete laboratory reporting* was available, the percentages of Black persons linked to care within 1 month of diagnosis (77.1%) and with viral suppression within 6 months of diagnosis (62.9%) during 2018 were lower than the Ending the HIV Epidemic initiative objectives of 95% for linkage to care and viral suppression goals (*2*). Access to HIV-related care and treatment services varies by residence area (*3**–**5*). Identifying urban-rural differences in HIV care outcomes is crucial for addressing HIV-related disparities among Black persons with HIV infection. CDC used National HIV Surveillance System^†^ (NHSS) data to describe HIV care outcomes among Black persons with diagnosed HIV infection during 2018 by population area of residence^§^ (area). During 2018, Black persons in rural areas received a higher percentage of late-stage diagnoses (25.2%) than did those in urban (21.9%) and metropolitan (19.0%) areas. Linkage to care within 1 month of diagnosis was similar across all areas, whereas viral suppression within 6 months of diagnosis was highest in metropolitan areas (63.8%). The Ending the HIV Epidemic initiative supports scalable, coordinated, and innovative efforts to increase HIV diagnosis, treatment, and prevention among populations disproportionately affected by or who are at higher risk for HIV infection (*6*), especially during syndemics (e.g. with coronavirus disease 2019). 

CDC analyzed data reported to NHSS for Black persons aged ≥13 years who received a diagnosis of HIV during 2018 in 41 states^¶ ^and the District of Columbia, jurisdictions in which laboratory reporting was complete as of December 31, 2019. Stage of disease^**^ at diagnosis was classified using the 2014 surveillance case definition for HIV infection based on age-specific CD4 counts or percentages of total lymphocytes (*2*,*7*). Linkage to care within 1 month of diagnosis was measured by documentation of one or more CD4 counts or percentage of viral load test results within 1 month after diagnosis. Viral suppression within 6 months of HIV diagnosis was defined as a viral load of <200 HIV RNA copies/mL within 6 months of HIV diagnosis. Data were statistically adjusted by using multiple imputation techniques to account for missing HIV transmission categories (*8*). Analyses were conducted using SAS (version 9.4; SAS Institute). 

Among 14,502 Black persons who received a diagnosis of HIV infection during 2018, a total of 897 (6.2%) lived in a rural area, 1,920 (13.2%) lived in an urban area, and 11,685 (80.6%) lived in a metropolitan area. The percentage of Black persons who received a late (stage 3, acquired immunodeficiency syndrome) diagnosis of HIV infection was highest in rural areas (25.2%), followed by urban and metropolitan areas (21.9% and 19.0%, respectively) (Table 1) (Supplementary Figure, https://stacks.cdc.gov/view/cdc/102576). Females were more likely than were males to receive a late-stage diagnosis. The highest percentage of late-stage diagnoses was among females in rural areas (females, rural: 31.4%, urban: 23.1%, metropolitan: 20.6%; males, rural: 23.0%, urban: 21.5%, metropolitan: 18.6%). The highest percentages of late-stage diagnoses occurred among persons aged 45–54 years in both rural and metropolitan areas (47.9% and 31.4%, respectively); in urban areas, the percentage of late-stage diagnoses was highest among persons aged ≥55 years (43.1%). By transmission category, the percentage of late-stage diagnoses was highest in all areas among males whose infection was attributed to heterosexual contact (rural: 37.2%, urban: 32.5%, metropolitan: 28.3%).

**TABLE 1:** Stage of disease at time of human immunodeficiency virus (HIV) diagnosis during 2018 among Black or African American persons aged ≥13 years, by population of area of residence and selected characteristics — 41 states and the District of Columbia, 2018*

Characteristic	Total no.	No. (%)				
		Stage zero^†^	Stage 1 (CD4 ≥500 cells/*µ*L or ≥ 26%)	Stage 2 (CD4 = 200–499 cells/*µ*L or 14%–25%)	Stage 3 (AIDS) (OI or CD4 <200 cells/*µ*L or <14%)	Stage unknown^§^
**Rural**						
**Gender**						
Male	**648**	40 (6.2)	137 (21.1)	197 (30.4)	149 (23.0)	125 (19.3)
Female	**242**	8 (3.3)	70 (28.9)	58 (24.0)	76 (31.4)	30 (12.4)
Transgender^¶^	**7**	2 (28.6)	0 (—)	1 (14.3)	1 (14.3)	3 (42.9)
**Age group at diagnosis, yrs**						
13–24	**225**	25 (11.1)	49 (21.8)	86 (38.2)	19 (8.4)	46 (20.4)
25–34	**289**	18 (6.2)	73 (25.3)	85 (29.4)	58 (20.1)	55 (19.0)
35–44	**163**	5 (3.1)	39 (23.9)	48 (29.4)	47 (28.8)	24 (14.7)
45–54	**117**	2 (1.7)	23 (19.7)	16 (13.7)	56 (47.9)	20 (17.1)
≥55	**103**	0 (—)	23 (22.3)	21 (20.4)	46 (44.7)	13 (12.6)
Transmission category**						
Male-to-male sexual contact	**489**	38 (7.8)	95 (19.5)	164 (33.5)	94 (19.2)	98 (20.0)
Injection drug use						
Male	**33**	0 (—)	12 (37.8)	3 (9.5)	11 (32.9)	6 (18.8)
Female	**19**	1 (2.6)	6 (32.0)	5 (23.7)	7 (35.1)	1 (6.7)
Male-to-male sexual contact and injection drug use	**14**	1 (8.4)	5 (32.9)	2 (15.4)	1 (9.1)	5 (34.3)
Heterosexual contact^††^						
Male	**118**	2 (1.3)	24 (20.5)	29 (24.7)	44 (37.2)	19 (16.4)
Female	**222**	9 (3.8)	64 (28.7)	52 (23.6)	69 (30.9)	29 (12.9)
**Total^§§^**	**897**	**50 (5.6)**	**207 (23.1)**	**256 (28.5)**	**226 (25.2)**	**158 (17.6)**
**Urban**						
**Gender**						
Male	**1,399**	77 (5.5)	300 (21.4)	448 (32.0)	301 (21.5)	273 (19.5)
Female	**502**	15 (3.0)	143 (28.5)	154 (30.7)	116 (23.1)	74 (14.7)
Transgender^¶^	**19**	3 (15.8)	4 (21.1)	9 (47.4)	3 (15.8)	0 (—)
**Age group at diagnosis, yrs**						
13–24	**575**	48 (8.3)	137 (23.8)	209 (36.3)	52 (9.0)	129 (22.4)
25–34	**651**	29 (4.5)	169 (26.0)	218 (33.5)	129 (19.8)	106 (16.3)
35–44	**323**	8 (2.5)	79 (24.5)	89 (27.6)	96 (29.7)	51 (15.8)
45–54	**211**	8 (3.8)	34 (16.1)	57 (27.0)	74 (35.1)	38 (18.0)
≥55	**160**	2 (1.3)	28 (17.5)	38 (23.8)	69 (43.1)	23 (14.4)
Transmission category**						
Male-to-male sexual contact	**1,121**	68 (6.1)	254 (22.6)	370 (33.0)	218 (19.5)	211 (18.8)
Injection drug use						
Male	**45**	2 (4.0)	9 (19.7)	13 (28.7)	10 (21.5)	12 (26.0)
Female	**36**	1 (3.6)	9 (26.2)	6 (16.7)	10 (28.4)	9 (25.1)
Male-to-male sexual contact and injection drug use	**28**	1 (4.7)	7 (25.4)	8 (29.7)	4 (15.6)	7 (24.6)
**Heterosexual contact^††^**						
Male	**221**	8 (3.4)	32 (14.5)	66 (29.8)	72 (32.5)	44 (19.8)
Female	**464**	15 (3.2)	134 (28.7)	147 (31.6)	105 (22.7)	64 (13.8)
**Total^§§^**	**1,920**	**95 (4.9)**	**447 (23.3)**	**611 (31.8)**	**420 (21.9)**	**347 (18.1)**
**Metropolitan**						
**Gender**						
Male	**8,502**	619 (7.3)	1,979 (23.3)	2,647 (31.1)	1,584 (18.6)	1,673 (19.7)
Female	**2,941**	119 (4.0)	912 (31.0)	817 (27.8)	606 (20.6)	487 (16.6)
Transgender^¶^	**242**	24 (9.9)	78 (32.2)	74 (30.6)	29 (12.0)	37 (15.3)
**Age group at diagnosis, yrs**						
13–24	**2,916**	292 (10.0)	760 (26.1)	1,029 (35.3)	273 (9.4)	562 (19.3)
25–34	**4,172**	294 (7.0)	1,129 (27.1)	1,289 (30.9)	667 (16.0)	793 (19.0)
35–44	**1,980**	83 (4.2)	492 (24.8)	563 (28.4)	469 (23.7)	373 (18.8)
45–54	**1,444**	56 (3.9)	309 (21.4)	351 (24.3)	453 (31.4)	275 (19.0)
≥55	**1,173**	37 (3.2)	279 (23.8)	306 (26.1)	357 (30.4)	194 (16.5)
Transmission category**						
Male-to-male sexual contact	**6,998**	567 (8.1)	1,702 (24.3)	2,225 (31.8)	1,157 (16.5)	1,347 (19.2)
Injection drug use						
Male	**283**	16 (5.6)	64 (22.4)	73 (25.6)	69 (24.4)	62 (21.9)
Female	**214**	10 (4.5)	58 (27.1)	55 (25.5)	50 (23.2)	42 (19.7)
Male-to-male sexual contact and injection drug use	**195**	14 (7.4)	51 (26.3)	52 (26.8)	34 (17.2)	44 (22.3)
Heterosexual contact^††^						
Male	**1,244**	44 (3.6)	233 (18.7)	363 (29.2)	352 (28.3)	252 (20.3)
Female	**2,722**	110 (4.1)	854 (31.4)	762 (28.0)	551 (20.2)	445 (16.4)
**Total^§§^**	**11,685**	**762 (6.5)**	**2,969 (25.4)**	**3,538 (30.3)**	**2,219 (19.0)**	**2,197 (18.8)**

**Abbreviations:** CD4 = CD4+ T-lymphocyte count (cells/*µ*L) or percentage; OI = opportunistic infection (i.e., AIDS-defining condition).

* Stage of disease at diagnosis of HIV infection based on first CD4 test performed or documentation of an AIDS-defining condition ≤3 months after a diagnosis of HIV infection. Data are based on residence at time of diagnosis. Data not provided for states and associated counties that do not have laws requiring reporting of all CD4 and viral loads, or that have incomplete reporting of laboratory data to CDC. Areas without laws: Idaho, New Jersey, and Pennsylvania. Areas with incomplete reporting: Arizona, Arkansas, Connecticut, Kansas, Kentucky, Vermont, and Puerto Rico.

^†^ First positive HIV test result is within 6 months after a negative HIV test result. The diagnosis of an AIDS-defining condition or a low CD4 test result before the 6 months have elapsed does not change the stage from stage zero to stage 3.

^§^ Includes persons with no CD4 information.

^¶^ Transgender includes persons who identified as transgender male-to-female, transgender female-to-male, and additional gender identity. Data not displayed because the numbers were too small to be meaningful. “Transgender male-to-female” includes persons who were assigned "male" sex at birth but have ever identified as "female" gender. "Transgender female-to-male" includes persons who were assigned "female" sex at birth but have ever identified as "male" gender. Additional gender identity examples include "bigender," "gender queer," and "two-spirit.”

** Data have been statistically adjusted to account for missing transmission category; therefore, values might not sum to column subtotals and total. Data presented based on sex at birth and include transgender persons.

^††^ Heterosexual contact with a person known to have, or to be at high risk for, HIV infection.

^§§^ Includes persons whose infection was attributed to hemophilia, blood transfusion, or perinatal exposure, or whose risk factor was not reported or not identified. Data not displayed because the numbers were too small to be meaningful.

Overall, the percentage of Black persons with HIV infection diagnosed during 2018 who were linked to care within 1 month of diagnosis was 76.7% in rural areas, 77.0% in urban areas, and 77.2% in metropolitan areas (Table 2) (Supplementary Figure, https://stacks.cdc.gov/view/cdc/102576). Males were less likely than were females to be linked to care, regardless of area (males, rural: 75.2%, urban: 75.0%, metropolitan: 76.4%; females, rural: 81.8%, urban: 82.7%, metropolitan: 79.5%). Males aged 45–54 years in rural and urban areas with infection attributed to heterosexual contact (rural: 69.9%, urban: 67.1%) and males aged 13–24 years in metropolitan areas with infection attributed to heterosexual contact (62.3%) accounted for the lowest percentage of being linked to care compared with persons with other modes of transmission in those areas.

**TABLE 2:** Linkage to human immunodeficiency virus (HIV) medical care within 1 month of HIV diagnosis among Black or African American persons aged ≥13 years with HIV infection diagnosed during 2018, by population area of residence, age group, and selected characteristics — 41 states and the District of Columbia, 2018*

Characteristic	Age 13–24 yrs		Age 25–34 yrs		Age 35–44 yrs		Age 45–54 yrs		Age ≥55 yrs		Total	
	No.	No. linked (%)	No.	No. linked (%)	No.	No. linked (%)	No.	No. linked (%)	No.	No. linked (%)	No.	No. linked (%)
**Rural**												
**Gender**												
Male	187	136 (72.7)	225	166 (73.8)	101	80 (79.2)	73	53 (72.6)	62	52 (83.9)	**648**	**487 (75.2)**
Female	36	29 (80.6)	61	45 (73.8)	60	53 (88.3)	44	33 (75.0)	41	38 (92.7)	**242**	**198 (81.8)**
Transgender^†^	2	1 (50.0)	3	3 (100)	2	0 (—)	0	0 (—)	0	0 (—)	**7**	**3 (42.9)**
**Transmission category^§^**												
Male-to-male sexual contact	177	128 (72.4)	185	138 (74.4)	63	49 (78.1)	41	30 (72.9)	22	18 (82.4)	**489**	**364 (74.4)**
Injection drug use												
Male	1	1 (75.0)	7	7 (89.3)	13	9 (74.8)	7	6 (82.6)	5	5 (100)	**33**	**27 (81.8)**
Female	2	2 (95.8)	4	3 (79.5)	4	4 (94.9)	4	4 (87.8)	5	5 (92.2)	**19**	**17 (89.5)**
Male-to-male sexual contact and injection drug use	2	1 (41.7)	6	2 (37.3)	4	3 (86.1)	1	1 (54.5)	1	1 (92.3)	**14**	**8 (57.1)**
Heterosexual contact^¶^												
Male	9	7 (83.1)	28	21 (75.1)	24	18 (76.7)	24	17 (69.9)	33	28 (82.3)	**118**	**91 (77.1)**
Female	34	27 (79.4)	57	41 (71.5)	56	49 (87.8)	40	29 (73.7)	36	33 (92.8)	**222**	**179 (80.6)**
Total^**^	**225**	**166 (73.8)**	**289**	**213 (73.7)**	**163**	**133 (81.6)**	**117**	**86 (73.5)**	**103**	**90 (87.4)**	**897**	**688 (76.7)**
**Urban**												
**Gender**												
Male	482	347 (72.0)	492	361 (73.4)	192	155 (80.7)	135	102 (75.6)	98	84 (85.7)	**1,399**	**1,049 (75.0)**
Female	87	68 (78.2)	147	117 (79.6)	130	114 (87.7)	76	67 (88.2)	62	49 (79.0)	**502**	**415 (82.7)**
Transgender^†^	6	5 (83.3)	12	10 (90.9)	1	0 (—)	0	0 (—)	0	0 (—)	**19**	**15 (78.9)**
**Transmission category^§^**												
Male-to-male sexual contact	454	329 (72.4)	421	311 (73.9)	126	102 (80.7)	76	62 (81.7)	45	40 (88.7)	**1,121**	**843 (75.2)**
Injection drug use												
Male	4	2 (55.0)	9	5 (52.2)	12	8 (66.1)	10	6 (66.0)	9	7 (77.4)	**45**	**29 (64.4)**
Female	6	4 (64.9)	6	5 (83.9)	10	8 (75.8)	7	6 (90.1)	8	6 (78.9)	**36**	**28 (77.8)**
Male-to-male sexual contact and injection drug use	6	4 (64.9)	9	7 (76.1)	5	3 (66.7)	6	4 (73.2)	2	1 (75.0)	**28**	**20 (71.4)**
Heterosexual contact^¶^												
Male	22	15 (67.7)	64	47 (74.0)	50	42 (84.0)	44	29 (67.1)	42	36 (84.7)	**221**	**168 (76.0)**
Female	80	63 (79.0)	142	113 (79.6)	120	106 (88.7)	69	61 (88.0)	54	43 (79.0)	**464**	**386 (83.2)**
**Total** ^**^	**575**	**420 (73.0)**	**651**	**488 (75.0)**	**323**	**269 (83.3)**	**211**	**169 (80.1)**	**160**	**133 (83.1)**	**1,920**	**1,479 (77.0)**
**Metropolitan**												
**Gender**												
Male	2,409	1,833 (76.1)	3,328	2,535 (76.2)	1,256	976 (77.7)	857	637 (74.3)	652	511 (78.4)	**8,502**	**6,492 (76.4)**
Female	412	315 (76.5)	736	579 (78.7)	692	552 (79.8)	581	460 (79.2)	520	431 (82.9)	**2,941**	**2,337 (79.5)**
Transgender**^†^**	95	74 (77.9)	108	81 (80.2)	32	29 (90.6)	6	5 (83.3)	1	1 (100)	**242**	**195 (80.6)**
**Transmission category^§^**												
Male-to-male sexual contact	2,339	0 (—)	2,941	2,261 (76.9)	921	722 (78.4)	509	374 (73.4)	288	224 (77.7)	**6,998**	**5,379 (76.9)**
Injection drug use												
Male	20	13 (63.6)	61	44 (71.5)	62	48 (76.6)	62	45 (71.9)	78	63 (80.1)	**283**	**211 (74.5)**
Female	18	14 (78.3)	49	34 (69.0)	45	32 (72.3)	45	35 (77.9)	56	46 (81.1)	**214**	**162 (75.7)**
Male-to-male sexual contact and injection drug use	37	29 (77.5)	92	71 (76.8)	33	23 (71.8)	20	15 (77.3)	14	10 (76.5)	**195**	**149 (76.4)**
Heterosexual contact^¶^												
Male	102	64 (62.3)	333	240 (72.0)	268	208 (77.5)	271	208 (76.8)	270	212 (78.7)	**1,244**	**931 (74.9)**
Female	386	292 (75.7)	688	545 (79.2)	649	522 (80.4)	535	424 (79.3)	463	385 (83.1)	**2,722**	**2,168 (79.6)**
Total**	**2,916**	**2,222 (76.2)**	**4,172**	**3,200 (76.7)**	**1,980**	**1,557 (78.6)**	**1,444**	**1,102 (76.3)**	**1,173**	**943 (80.4)**	**11,685**	**9,024 (77.2)**

**Abbreviations:** CD4 = CD4+ T-lymphocyte count (cells/*µ*L) or percentage; OI = opportunistic infection (i.e., AIDS-defining condition).

* Linkage to HIV medical care was measured by documentation of ≥1 CD4 or VL tests ≤1 month or ≤3 months of HIV diagnosis. Data are based on residence at time of diagnosis. Data not provided for states and associated counties that do not have laws requiring reporting of all CD4 and viral loads, or that have incomplete reporting of laboratory data to CDC. Areas without laws: Idaho, New Jersey, and Pennsylvania. Areas with incomplete reporting: Arizona, Arkansas, Connecticut, Kansas, Kentucky, Vermont, and Puerto Rico.

^†^ Transgender includes persons who identified as transgender male-to-female, transgender female-to-male, and additional gender identity. Data not displayed because the numbers were too small to be meaningful. “Transgender male-to-female” includes persons who were assigned "male" sex at birth but have ever identified as "female" gender. "Transgender female-to-male" includes persons who were assigned "female" sex at birth but have ever identified as "male" gender. Additional gender identity examples include "bigender," "gender queer," and "two-spirit.”

^§^ Data have been statistically adjusted to account for missing transmission category; therefore, values might not sum to column subtotals and total. Data presented based on sex at birth and include transgender persons.

^¶^ Heterosexual contact with a person known to have, or to be at high risk for, HIV infection.

** Includes persons whose infection was attributed to hemophilia, blood transfusion, perinatal exposure, or whose risk factor was not reported or not identified. Data not displayed because the numbers were too small to be meaningful.

Overall, the percentage of Black persons aged ≥13 years in rural areas with HIV diagnosed during 2018 who had <200 copies of viral RNA per mL (viral suppression) within 6 months of diagnosis was 59.6% in rural areas, 59.7% in urban areas, and 63.8% in metropolitan areas (Table 3) (Supplementary Figure, https://stacks.cdc.gov/view/cdc/102576). The percentage of males with viral suppression within 6 months of diagnosis was lower than the percentage among females, regardless of area (males, rural: 58.0%, urban: 57.8%, metropolitan: 62.4%; females, rural: 64.0%, urban: 65.1%, metropolitan: 68.1%). By age group and area, the lowest percentage of viral suppression within 6 months of diagnosis was among persons aged 45–54 years in rural and urban areas (52.1% and 56.4%, respectively) and persons aged 13–34 years in metropolitan areas (62.6%). In rural and urban areas, the lowest percentage of viral suppression within 6 months of diagnosis was among males aged 45–54 years with infection attributed to male-to-male sexual contact and to heterosexual contact (44.2% and 42.5%, respectively). In metropolitan areas, the lowest percentage of viral suppression within 6 months of diagnosis was among males aged 13–24 years with infection attributed to heterosexual contact (51.7%) and males aged 25–34 years with infection attributed to injection drug use (IDU) (45.0%).

**TABLE 3:** Human immunodeficiency virus (HIV) viral suppression within 6 months among Black or African American persons aged ≥13 years with HIV infection diagnosed during 2018, by population area of residence, age group, and selected characteristics — 41 states and the District of Columbia, 2018*

Characteristic	Age ≥13 yrs		Age 13–24 yrs		Age 25–34 yrs		Age 35–44 yrs		Age 45–54 yrs		Age ≥55 yrs	
	No.	Suppressed no. (%)	No.	Suppressed no. (%)	No.	Suppressed no. (%)	No.	Suppressed no. (%)	No.	Suppressed no. (%)	No.	Suppressed no (%)
**Rural**												
**Gender**												
Male	648	376 (58.0)	187	118 (63.1)	225	132 (58.7)	101	61 (60.4)	73	33 (45.2)	62	32 (51.6)
Female	242	155 (64.0)	36	22 (61.1)	61	33 (54.1)	60	42 (70.0)	44	28 (63.6)	41	30 (73.2)
Transgender^†^	7	4 (57.1)	2	0 (—)	3	2 (66.7)	2	2 (100)	0	0 (—)	0	0 (—)
**Transmission category^§^**												
Male-to-male sexual contact	489	282 (57.6)	177	110 (62.0)	185	111 (60.0)	63	33 (52.3)	41	18 (44.2)	22	10 (43.4)
Injection drug use												
Male	33	18 (55.1)	1	1 (75.0)	7	4 (48.0)	13	10 (75.6)	7	2 (31.9)	5	2 (41.3)
Female	19	12 (60.8)	2	2 (79.2)	4	2 (51.3)	4	3 (79.6)	4	3 (61.0)	5	2 (45.1)
Male-to-male sexual contact and injection drug use	14	9 (61.5)	2	1 (29.2)	6	4 (62.7)	4	3 (75.0)	1	0 (—)	1	1 (100)
Heterosexual contact^¶^												
Male	118	71 (60.5)	9	7 (79.8)	28	15 (54.4)	24	18 (75.0)	24	12 (51.3)	33	19 (56.6)
Female	222	142 (63.8)	34	20 (79.2)	57	30 (52.2)	56	39 (69.2)	40	26 (63.9)	36	28 (77.2)
Total**	**897**	**535 (59.6)**	**225**	**140 (62.2)**	**289**	**167 (57.8)**	**163**	**105 (64.4)**	**117**	**61 (52.1)**	**103**	**62 (60.2)**
**Urban**												
**Gender**												
Male	1,399	808 (57.8)	482	275 (57.1)	492	286 (58.1)	192	118 (61.5)	135	73 (54.1)	98	56 (57.1)
Female	502	327 (65.1)	87	56 (64.4)	147	99 (67.3)	130	88 (67.7)	76	46 (60.5)	62	38 (61.3)
Transgender^†^	19	11 (57.9)	6	4 (66.7)	12	7 (58.3)	1	0 (—)	0	0 (—)	0	0 (—)
**Transmission category^§^**												
Male-to-male sexual contact	1,121	662 (59.1)	454	259 (57.2)	421	250 (59.5)	126	82 (65.3)	76	44 (58.0)	45	26 (58.1)
Injection drug use												
Male	45	22 (48.9)	4	2 (45.0)	9	3 (29.3)	12	6 (51.6)	10	7 (67.0)	9	4 (47.3)
Female	36	20 (56.0)	6	4 (73.7)	6	4 (67.9)	10	6 (56.6)	7	3 (47.9)	8	3 (40.8)
Male-to-male sexual contact and injection drug use	28	14 (50.0)	6	4 (56.3)	9	5 (51.1)	5	1 (16.7)	6	4 (67.9)	2	1 (56.3)
Heterosexual contact^¶^												
Male	221	118 (53.3)	22	12 (55.3)	64	34 (53.6)	50	29 (57.1)	44	19 (42.5)	42	24 (58.2)
Female	464	307 (66.0)	80	52 (64.7)	142	95 (67.4)	120	82 (68.6)	69	43 (61.8)	54	35 (64.1)
**Total** ^**^	**1,920**	**1,146 (59.7)**	**575**	**335 (58.3)**	**651**	**392 (60.2)**	**323**	**206 (63.8)**	**211**	**119 (56.4)**	**160**	**94 (58.8)**
**Metropolitan**												
**Gender**												
Male	8,502	5,301 (62.4)	2,409	1,503 (62.4)	3,328	2,067 (62.1)	1,256	796 (63.4)	857	534 (62.3)	652	401 (61.5)
Female	2,941	2,003 (68.1)	412	267 (64.8)	736	483 (65.6)	692	486 (70.2)	581	410 (70.6)	520	357 (68.7)
Transgender**^†^**	242	147 (60.7)	95	56 (58.9)	108	58 (53.7)	32	26 (81.3)	6	3 (50.0)	1	1 (100)
**Transmission category^§^**												
Male-to-male sexual contact	6,998	4,420 (63.2)	2,339	1,474 (63.0)	2,941	1,856 (63.1)	921	595 (64.6)	509	315 (62.0)	288	180 (62.3)
Injection drug use												
Male	283	146 (51.5)	20	7 (37.4)	61	28 (45.0)	62	32 (52.1)	62	31 (50.0)	78	47 (60.8)
Female	214	124 (58.0)	18	11 (61.4)	49	23 (47.5)	45	25 (54.9)	45	30 (65.3)	56	35 (62.8)
Male-to-male sexual contact and injection drug use	195	112 (57.4)	37	21 (55.5)	92	53 (57.8)	33	17 (52.1)	20	11 (57.1)	14	10 (72.8)
Heterosexual contact^¶^												
Male	1,244	756 (60.8)	102	53 (51.7)	333	188 (56.4)	268	174 (64.8)	271	179 (66.1)	270	163 (60.4)
Female	2,722	1,873 (68.8)	386	248 (64.4)	688	459 (66.7)	649	464 (71.4)	535	380 (71.0)	463	322 (69.4)
Total**	**11,685**	**7,451 (63.8)**	**2,916**	**1,826 (62.6)**	**4,172**	**2,611 (62.6)**	**1,980**	**1,308 (66.1)**	**1,444**	**947 (65.6)**	**1,173**	**759 (64.7)**

**Abbreviations:** CD4 = CD4+ T-lymphocyte count (cells/*µ*L) or percentage; OI = opportunistic infection (i.e., AIDS-defining condition); VL = viral load.

* VL test result of <200 copies/ml indicates HIV viral suppression. VL test results are within 6 months of diagnosis of HIV infection during 2018. Data are based on residence at time of diagnosis. Data not provided for states and associated counties that do not have laws requiring reporting of all CD4 and VLs, or that have incomplete reporting of laboratory data to CDC. Areas without laws: Idaho, New Jersey, and Pennsylvania. Areas with incomplete reporting: Arizona, Arkansas, Connecticut, Kansas, Kentucky, Vermont, and Puerto Rico.

^†^ Transgender includes persons who identified as transgender male-to-female, transgender female-to-male, and additional gender identity. Data not displayed because the numbers were too small to be meaningful. “Transgender male-to-female” includes persons who were assigned "male" sex at birth but have ever identified as "female" gender. "Transgender female-to-male" includes persons who were assigned "female" sex at birth but have ever identified as "male" gender. Additional gender identity examples include "bigender," "gender queer," and "two-spirit.”

^§^ Data have been statistically adjusted to account for missing transmission category; therefore, values might not sum to column subtotals and total. Data presented based on sex at birth and include transgender persons.

^¶^ Heterosexual contact with a person known to have, or to be at high risk for, HIV infection.

## Discussion

During 2018, one in four (25.2%) diagnosed HIV infections among Black persons in rural areas was a late-stage diagnosis, a percentage that was higher than that among Black persons in urban (21.9%) and metropolitan (19.0%) areas. The percentages of patients linked to care within 1 month of diagnosis were similar in all areas, whereas the percentages of persons with viral suppression within 6 months of diagnosis were lower in rural (59.6%) and urban (59.7%) areas than in metropolitan areas (63.8%). In all areas, the percentages of persons who were linked to care within 1 month of diagnosis and who had viral suppression within 6 months of diagnosis were substantially below the Ending the HIV Epidemic initiative targets of 95% (*9*). These findings likely underscore known differences in health-related behaviors, physical and sociocultural environments, and access to and use of health care systems among Black urban and rural HIV populations (*3*,*4*).

By transmission category, the highest percentages of late-stage diagnoses in all areas were among males with infection attributed to heterosexual contact. The lowest levels of linkage to care within 1 month of diagnosis were among males in rural areas with infection attributed to both male-to-male sexual contact and IDU, and males in urban areas with infection attributed to IDU. Viral suppression within 6 months of diagnosis was least common in all areas among males aged ≥13 years with infection attributed to IDU. Broader implementation of routine HIV testing is needed to identify persons with undiagnosed infections and to initiate early treatment, particularly among older persons. Interventions that support patient retention and re-engagement in HIV care are necessary to improve care outcomes and reduce HIV transmission. Locally tailored strategies among Black persons who inject drugs and sexually active adults at higher risk for HIV infection should be implemented for effective prevention in both urban and rural areas.

The findings in this report are subject to at least three limitations. First, analyses were limited to the 42 jurisdictions with complete laboratory reporting; these jurisdictions might not be representative of all Black persons living with diagnosed HIV infection in the United States. Second, CD4 and viral load test results reported to HIV surveillance programs were used for determining stage of disease and monitoring linkage to care and viral suppression; CD4 and viral load laboratory tests might not have been obtained at all care visits. Not having these tests performed among patients in care or unreported to surveillance systems limits the ability to monitor care outcomes. Finally, comparisons of numbers and percentages by area, sex, age group, and transmission category should be made cautiously because population subgroups vary in size and some have small numbers. Reported numbers ≤12 and their accompanying percentages are not discussed. 

Early HIV diagnosis and treatment among Black persons with HIV infection are necessary to reduce disparities and achieve national prevention goals. For equitable health to be achieved for Black persons in all geographic areas, culturally appropriate and stigma-free sexual health care is needed, particularly among those who live in rural communities. Although 80% of Black persons with diagnosed HIV live in metropolitan areas, identifying geographic disparities is important to ensure HIV-related health equity. Disparities in care outcomes should be addressed and interventions prioritized that address social determinants of health.^††^

**Summary**

**What is already known about this topic?**

Disparities in HIV care outcomes exist for Black persons with diagnosed human immunodeficiency virus (HIV) infection, and access to care and treatment services varies by residence area.

**What is added by this report?**

During 2018, rural Black persons received a higher percentage of late-stage HIV diagnosis (25.2%) than did those in urban (21.9%) and metropolitan areas (19.0%). Linkage to care within 1 month of diagnosis was similar across geographic areas; however, viral suppression within 6 months of diagnosis was highest in metropolitan areas (63.8%).

**What are the implications for public health practice?**

Early diagnosis and prompt treatment of Black persons with HIV infection, especially in rural areas, are necessary to reduce disparities in HIV care outcomes.

## Footnotes

* CDC has established three criteria for complete laboratory reporting: 1) the jurisdiction’s laws or regulations require reporting of all CD4 and viral load results to the state or local health department; 2) laboratories that perform HIV-related testing for the area must have reported a minimum of 95% of HIV-related test results to the state or local health department; and 3) by December 31, 2019, the jurisdiction had reported to CDC ≥95% of all CD4 and viral load results received during January 2017–September 2019. https://www.cdc.gov/hiv/library/reports/hiv-surveillance.html

† The National HIV Surveillance System is the primary source for monitoring HIV trends in the United States. Through that system, CDC funds and assists state and local health departments for collecting data regarding HIV infection cases. Health departments provide deidentified data to CDC.

§ Area of residence at HIV diagnosis was categorized as rural (<50,000 population), urban (50,000–499,999 population), or metropolitan (≥500,000 population) according to the Office of Management and Budget 2010 standards for delineating metropolitan and micropolitan statistical areas (http://www.federalregister.gov/documents/201006/28/2010-15605/2010-standards-for-delineating-metropolitan-and-micropolitan-statistical-areas).

¶ Alabama, Alaska, California, Colorado, Delaware, Florida, Georgia, Hawaii, Illinois, Indiana, Iowa, Louisiana, Maine, Maryland, Massachusetts, Michigan, Minnesota, Mississippi, Missouri, Montana, Nebraska, Nevada, New Hampshire, New Mexico, New York, North Carolina, North Dakota, Ohio, Oklahoma, Oregon, Rhode Island, South Carolina, South Dakota, Tennessee, Texas, Utah, Virginia, Washington, West Virginia, Wisconsin, and Wyoming.

** Disease stages are defined as follows: stage zero, the first positive HIV test result ≤6 months after a negative HIV test result and remaining at Stage zero until 6 months after the first positive test result; stage 1, a CD4 count of ≥500 cells/*µ*L or CD4 percentage of ≥26; stage 2, a CD4 count of 200–499 cells/*µ*L or CD4 percentage of 14–25; stage 3 (acquired immunodeficiency syndrome [AIDS]), a CD4 count of <200 cells/*µ*L or CD4 percentage of <14 or documentation of an AIDS-defining condition. Stages of disease are further classified as stage zero; stages 1–2: early-stage diagnosis; and stage 3 [AIDS]: late-stage diagnosis.
